# Situational analysis of the quality of palliative care services across India: a cross-sectional survey

**DOI:** 10.3332/ecancer.2022.1486

**Published:** 2022-12-08

**Authors:** Arunangshu Ghoshal, Anjum Khan Joad, Odette Spruijt, Shobha Nair, MR Rajagopal, Firuza Patel, Anuja Damani, Jayita Deodhar, Dinesh Goswami, Geeta Joshi, Savita Butola, Charu Singh, Seema Rajesh Rao, Madhura Bhatwadekar, Mary Ann Muckaden, Sushma Bhatnagar

**Affiliations:** 1 Department of Palliative Medicine, Tata Memorial Center, Homi Bhabha National Institute, Mumbai 400012, India; 2 Department of Anaesthesia and Palliative Care Medicine, Bhagwan Mahaveer Cancer Hospital and Research Center, Jaipur 302017, India; 3 Faculty of Medicine, Dentistry and Health Sciences, University of Melbourne, Melbourne, Victoria 3010, Australia; 4 Department of Palliative Medicine, Amrita Hospital, Kochi 682041, India; 5 Trivandrum Institute of Palliative Sciences and Pallium India, Aisha Memorial Hospital Building, Paruthikuzhy, Thiruvananthapuram 695009, India; 6 Department of Radiotherapy, Post Graduate Institute of Medical Education and Research, Chandigarh 160012, India; 7 Guwahati Pain and Palliative Care Society, Uzan Bazar, Guwahati, Assam 781001, India; 8 Community Oncology Center, Gujarat Cancer Society, Ahmedabad 380007, India; 9 Border Security Force Sector Hospital, Panisagar, Tripura 799260, India; 10 BHT Karunashraya, Bengaluru 560037, India; 11 Palliative Care Social Worker, Pune, Maharashtra 411058, India; 12 Department of Onco-Anaesthesia and Palliative Medicine, Dr. B.R.A Institute Rotary Cancer Hospital, All India Institute of Medical Sciences, New Delhi 110029, India

**Keywords:** palliative care, India, survey, quality improvement

## Abstract

**Objective:**

Palliative care services in India were established in the 1980s but there is no detailed up-to-date knowledge about the quality-of-service provision nationally. We aim to describe the current quality of palliative care provision in India, as measured against nationally adopted standards.

**Method:**

A digital survey adapted from the Indian Association of Palliative Care Standards Audit Tool was administered to 250 palliative care centres.

**Results:**

Two hundred and twenty-three (89%) palliative care centres participated – 26.4% were government-run, while the rest include non-governmental organisations, private hospitals, community-led initiatives and hospices. About 200 centres ‘often’ or ‘always’ fulfilled 16/21 desirable criteria; however, only 2/15 essential criteria were ‘often’ or ‘always’ fulfilled. Only 5.8% provide uninterrupted access to oral morphine.

**Significance of the results:**

Palliative care centres in India are falling short of meeting the essential quality standards, indicating the urgent need for new initiatives to drive national change.

## Key message

An 87% global increase in serious health-related suffering amenable to palliative care interventions is predicted by 2060. Despite an increasing need, palliative care is not reaching the required levels; at least in India, the second most populated country with a sixth of the world’s population. Analysing with the Indian Association of Palliative Care national quality standards reveals gaps in the provision of quality palliative care across India. The failure of most services to meet the essential criteria of uninterrupted supply of morphine, and comprehensive assessment and documentation, including prescribed opioids, needs to be addressed urgently. Identification of government and other key stakeholders to assist with the development of the palliative care workforce is much needed to enhance the efforts of the palliative care community.

*‘… by focusing on the most marginalized and excluded, we just might create a system that is, in fact, better for all of us’ Kedar Mate 2021 ED MANAGEMENT Vol. 33, No. 3; p. 25-36.*

## Background and rationale

Specialist palliative care is available for only 14% of the global population [1]. Worldwide, over 56.8 million people are estimated to require palliative care annually, including 25.7 million near the end of life. In India, the estimated populations in need are 5.5 million and 4.5 million, respectively.^1^ The Lancet Commission report estimates that 7 million Indians need palliative care [4].

An ageing population and increasing morbidity and mortality from cancer and other non-communicable diseases have created an urgent imperative to ensure adequate palliative care provision. Also, the mortality rates of cancer in India and other low-and-middle-income countries (LMICs) are higher because of the late presentation of patients, the high cost of health care (which is largely borne by patients and families) and the limited availability of treatments [2]. Limited infrastructure, large population, complex geography and poverty are some of the known challenges for healthcare policy implementation [3].

Globally, patients with HIV and cancer are the two largest disease groups requiring adult palliative care [4]. More than 1 million new cancer cases are diagnosed each year in India [5] of which 80% of patients present with stage III or stage IV disease [6]. The estimated need for palliative care for patients with a cancer diagnosis in India is 70–140 per 100,000 population [7]. Universal access to palliative care is recommended by key international bodies to relieve patients’ suffering [8] but less than 4% of Indians have access to this form of healthcare [9].

There is almost no data about the quality of palliative care services across India. In both Quality of Death Index studies [10, 11], dying in India was rated at the lower end of the scale; many gaps have been identified including inaccessibility to essential opioid analgesics such as morphine [4, 12, 13]. As per the Narcotic Drugs and Psychotropic Substances Act (Amendment) 2015, any registered medical practitioner in India can prescribe opioids, if they have training in pain relief and palliative care or opioid substitution therapy for treating opioid dependence [14]. However, while access to opioids is used as a surrogate indicator of access to palliative care, estimates of Serious Health-Related Suffering and the proposed metric of suffering intensity-adjusted life years are newer methods of comprehensively estimating palliative care needs [4]. Likewise, measuring the quality of care is necessary to evaluate the impact on relief of suffering from currently available services.

The Indian Association of Palliative Care (IAPC) is a national forum that aims to connect, support and motivate individuals/ institutions in palliative care. The IAPC was set up on 16 March 1994, in consultation with the World Health Organization (WHO) and the Government of India. The centres listed on the IAPC website are centres providing Palliative Care in India [15]. In 2008, the IAPC developed a National Quality Standards Audit Tool for palliative care services in India to drive quality improvement [16]. This audit tool is divided into ‘Essential’ and ‘Desirable’ sections, which are each further divided into five and seven sections, respectively. The essential components relate to documentation, essential medicines, training of team members in palliative care practice, degree of integration with the community and team professional development. Desirable components relate to programme features including providing access to free morphine and palliative drugs; an organisational culture that promotes self-care and is supported in the wider organisation; education and training; and quality improvement. Auditing services using these IAPC Standards Audit Tool components, therefore, provides a detailed understanding of the status of palliative care service provision in India.

## Methodology

This audit project was a descriptive cross-sectional survey.

*Development of the survey questionnaire*

The survey questionnaire was derived from the IAPC Standards Audit Tool version 2018. The development of the IAPC Standards Audit Tool has been described elsewhere [16]. The questionnaire requires 15–20 minutes for administration. Survey respondents are asked to rate their compliance with each standard, using scoring of ‘never’, ‘rarely’, ‘sometimes’, ‘often’ and ‘always’.

### Recruitment

All palliative care centres registered with the IAPC were eligible for recruitment. Informed consent forms were emailed to all the eligible centres before initiating the survey through Google Forms [17]. Participation in this survey was voluntary, and all responses were anonymised. Each centre completed one survey questionnaire. The research assistant contacted the designated person/representative of the participating centres from the details available on the IAPC website. In the absence of a designated palliative care physician in the centre, a senior employee/trustee well-acquainted with the service was asked to complete the questionnaire. In case of non-completion, the research assistant helped in the completion of the survey questionnaire through a phone follow-up at a regular interval of 2 weeks. The survey was conducted between January 2019 and June 2019. The Institutional Ethics Committee at The Tata Memorial Center (IEC/0718/2052/001) exempted this project from review.

### Statistical analysis

Our primary objective was to evaluate the achievement of Essential Criteria (See Appendix). We summarised the availability of various types of palliative care services at different centres using descriptive statistics, including means, proportions and frequencies, where appropriate. Data were analysed using Statistical Package for the Social Sciences version 27.0 [18].

## Results

### Type of services

Two hundred and fifty centres were sent the survey, out of which 223 (89%) responded. Centres that did not complete the questionnaire after two reminders (27) were considered non-responders. Nine (4%) respondents were senior employees/trustees of the service. The 223 services were mainly concentrated in large cities and regional cancer centres, except for Kerala, where services were more widespread (Table 1).

### Palliative care practice

Palliative care provision for patients with cancer and non-cancer diagnoses was the central service activity for only 85 (38%) of responding centres, whereas the rest follow a mixed practice model combining palliative care with other aspects of practice. The number of patients served ranged from an annual census of 30 patients in the smallest service to around 6,000 patients in the largest service (Table 1).

### ‘Essential’ palliative care components

#### The hospices and palliative care centres met the essential standards as follows:

Adherence to whole patient assessment, documentation and management was not uniform. The three subcategories of symptom assessment and documentation, namely pain scale, other symptoms and regular review of symptoms and titration of medicines, tended to be completed ‘sometimes’ in 89%, 89% and 86% centres, respectively. Access to essential medicines and equipment (Essential Package) was provided ‘always’ by 89% of respondents; but when narrowed down, we found that only 5.8% had an uninterrupted supply of immediate-release oral morphine. A system for documentation of step 3 opioids was possible only ‘sometimes’ in 89% of responding services. A trained doctor and nurse, defined by IAPC as having a minimum of 10 days of supervised clinical palliative care training, were available ‘sometimes’ in 85% and 83% of services, respectively; both were ‘always’ available in 5% of services. Designated team members trained to deliver psychological, social and spiritual support were also available only ‘sometimes’ in 86% of cases (Table 1).

Interaction between the community and other health care professionals in the establishment and ongoing operation of the services was evident ‘sometimes’ in 88% of centres. Regular team meetings occurred ‘sometimes’ in 82%, and self-care training ‘sometimes’ in 86% of respondents. Debriefing was done only rarely by most respondents (86%). While 89% of centres ‘often’ conducted educational programmes on palliative care for fellow professionals, 89% of centres ‘rarely’ organised ongoing continuing professional education for the palliative care team.

### ‘Desirable’ palliative care components

#### Respondents met the desirable standards as follows:

Most desirable standards’ domains were ‘often’ met including pain policies (87%), end-of-life care (89%), access to ancillary services (dietetics, physiotherapy, occupational therapy) (89%), caregiver support (87%), allied health support (89%), advocacy and awareness-raising programmes (88%) and regular team building (88%) (Table 1).

Significant volunteer contributions were ‘often’ met by 64% of respondents and ‘never’ by 25% of respondents. However, 87% of respondents only ‘sometimes’ had sufficient access to free morphine (an essential package for poor patients), provided home care services directly/indirectly (92.3%) and had access to after-hours support (directly/indirectly) (90%). Other desirable standards were met most of the time by most services.

## Discussion

Our survey provides detailed information on the functioning of 223 palliative care services in India in 2019. This paper reports the second round of audit cycle using this tool, the first being in 2008 [16]. There was considerable variation associated with the type of service and size of the annual patient population. Most services met both essential and desirable standards only ‘sometimes’. Only 5% of services were consistently able to provide an uninterrupted supply of oral morphine for their patients.

### The high response rate in this survey

The high response rate of 83% of surveyed services is notable compared with a recent mapping survey of US palliative care services, which had a 69% response rate [19, 20]. The number of participating centres also increased compared to previous mapping surveys conducted in India – 138 hospice and palliative care services in 2007 [21], 49 palliative care organisations in 2008 [16] and 102 centres listed with the National Cancer Grid providing cancer palliative care in 2008, participated in mapping service levels [22]. The high response rate in this current survey may be an indication of the high level of engagement with quality and service improvement in Indian palliative care, as led by the IAPC and the recent EQuIP (Enable Quality, Improve Patient Care) India programme conducted through the National Cancer Grid. Also, reminding the respondents with phone calls at regular intervals might have contributed to the high response rate, as has been seen elsewhere [23]. Similar survey tools to assess facilitators and barriers to quality measurement and improvement in palliative care programmes have been developed in the USA on a convenience sample of 11 palliative care programmes with a five-part, adaptable, modular survey addressing barriers and facilitators to quality initiatives within palliative care teams [24]. Quality assessment is an integral part of palliative care programmes in countries where palliative care is more developed [10, 25–27]. The tools developed are aligned with those used in our audit. A strength of this audit is that the tool is designed specifically for the Indian context and as such, may be of value for countries at a similar stage of development of palliative care.

### Significance of our results

The delivery of palliative care is seen increasingly as a global health issue and has been highlighted widely by the World Health Assembly declaration in 2014 [28], the Lancet Commission Report on Palliative Care and Pain Relief ‘serious health-related suffering’ construct in 2017 [4] and by the Declaration of Astana, focusing on primary care as an aspect of Universal Health Coverage and sustainable development goals, in 2018 [29]. Unfortunately, the estimated burden of serious health-related suffering will almost double by 2060, with the fastest increases occurring in low-income countries [4]. It is thus essential to portray the palliative care services from India, which is the second most populated country in the world with a sixth of the world’s population [30].

Our results suggest that there is wide variation in the functioning of services throughout India. However, it is a matter of concern that less than 20 services often/always meet the ‘Essential’ criteria of the IAPC national standards. It might also be regarded as a critical concern that more than 80% of participating palliative care centres across India cannot guarantee their patients an uninterrupted supply of oral morphine. The availability of morphine is linked to the level of integration of palliative care into a country’s health care system and the achievement of standards of health, education and income [31]. WHO Guidelines (2000, 2011) emphasise the quadruple imperative: public health, moral, legal and political, to make morphine accessible and available [32]. The availability and consumption of morphine and/or its equivalents are grossly inadequate in India, with only 43 mg per patient in need of palliative care, compared to high-income and upper-middle-income countries [4, 33]. Improving access to oral morphine and implementation of the Narcotic Drugs and Psychotropic Substances (Amendment) Act, 2015 must continue to be a priority for the IAPC and national policymakers and those that are charged with implementing the amended Act at the state level [34, 35].

### Characteristics of the services and standards

**Community versus hospital** This survey demonstrated that the capacity to meet the standards varied according to provider type. More community-based providers^2^ [36] reported ‘always’ on all the essential measures than hospital-based providers, except for assessment and documentation of pain, and a system of documentation of step 3 opioids. Hospital-based providers scored higher than community-based providers in the essential domains of professional development and self-care. These differences suggest the different settings have different logistical and operational priorities and capabilities. Hospital-based providers may place more emphasis on and have more resources dedicated to, developing and adhering to standard operating procedures, and accreditation with national boards/state government boards. Community-based providers such as in Kerala may rely more heavily on volunteerism and have more direct engagement with the community they serve, leading to prioritisation of the clinical and social needs of their patients. Only 26% of participating centres were in the government sector indicating a large gap in the provision of palliative care to patients from lower socioeconomic groups, which constitute much of the Indian population cared for in this sector. Unless there is an intensive rollout of palliative care into the government sector, the unnecessary suffering of these patients will remain unrelieved.

**Programme size** also appeared to impact the priorities of the services. Programmes with more than 750 patients annually like the Regional Cancer Centers had better systems in place for the documentation of pain and other symptoms and scored highest on availability of funds, professional staff development and participation in research. Programmes with around 100–150 patients annually reported greater community support and reported better organisational health, support from volunteers and administrators, conflict resolution, debriefing and ongoing audits. These differences are possibly due to the larger centres being established for a longer duration which provided them the time to develop stronger infrastructure and secure funding. The high-volume centres, unfortunately, were not necessarily able to provide essential opioid medications for free, in contrast to much smaller centres in the community due to differences in service delivery model. In this survey, home care services were provided directly/indirectly by 92.3% of centres surveyed. International literature suggests that home-based palliative care services may be offered as part of a geriatrics or primary care practice with expertise in palliative care (primary and palliative care), via integration of a speciality practice of palliative care, or through home care (home health), hospice agencies or a hospital-based palliative care programme. The funding and coverage of such home care varies throughout the world, but generally coverage is higher in developed nations compared to others [37].

### Suggested solutions

#### Education and training in palliative care

Education and training are critical to quality improvement. Although 68% of centres were focused on providing palliative care, this project identified that many lack trained palliative care specialists or training programmes. The few specialists who graduate through the MD programme each year [38] will not be able to fulfil these lacunae in the foreseeable future. Opportunities to participate in palliative care training modules and clinical rotations should be an essential component in the training curriculum of all physicians. Mexico provides an example of the national prioritisation of competencies in palliative care. In 2007/2008, Mexico legislated that all health care personnel must have palliative care training and all health care institutions must ‘guarantee the training and continued education of human resources in health care in the area of palliative care and attention to patients in the terminal phase’ [39]. Improvement in service provision can only be achieved by the combined efforts of all relevant stakeholders. Administrators need to ensure that all the health care workers in their service have ongoing access to the recommended levels of training and clinical exposure to palliative care practice and to provide continuous training and quality improvement/audit opportunities. Only nine centres offered ongoing education for their palliative care teams. Hospitals must employ palliative care trained health care staff and provide continuous professional development opportunities for these teams. With increased training, services are more likely to improve the comprehensiveness of patient assessment and documentation which was a poor performing standard in this audit. Most teams did not have designated team members for psychological, social and spiritual support. Expanding these domains of service provision by employing designated team members would also help sustain the palliative care workforce by providing more opportunities for debriefing and self-care and improving the ability to meet the national standards and the overall quality of care. This workforce can only be generated if palliative care is a part of the undergraduate and postgraduate curriculum of mental health and social work professionals. National measures to enable the rollout of critical aspects of the essential package such as the uninterrupted supply of essential controlled medications like morphine are needed, rather than the current reliance on individual services to organise these packages. Team self-care and health are another area that needs to be prioritised by hospital authorities and management alike and to be recognised as an essential component of palliative care service sustainability.

## Role of IAPC in driving change

The IAPC has a major role to play in driving such service improvement across India, in providing advocacy leadership at the central and state government levels, and in working with non-government partners to drive change. International examples of such leadership include the National Quality Forum in the US [40], and the National Standards for Providing Palliative Care programme of Australia [41]. Previously, Australian services were stratified into four levels, from primary care providers of palliative care to increasingly more specialist and academic services/departments [42]. However, in the 2018 Australian standards [41], there has been a move away from this stratification concept, to a more generic, normative, articulation of the principles of providing best practice palliative care, principles which are relevant to all services, regardless of team size, location and resources. The IAPC standards already recognise the aspirational dimension of standards, as well as the essential components of service provision, and thus provide Indian services with a roadmap to guide their development. As such, the IAPC standards are more aligned with this latest version of the Australian standards. Ongoing clarification of the core principles underlying these standards may further guide services in their efforts to improve the quality of care. Establishing the capacity for a service self-assessment programme within an IAPC national standards programme could also help to foster ongoing development and quality improvement.

## Importance of continuity of care, links across service settings, integration

There is a need for greater integration of palliative care and better continuity of care across settings of care, from outpatient department to inpatient to community-based care. Many services are limited to one set of care. Only 68% provided inpatient services. Patients with more complex needs may need access to an inpatient palliative care facility. Similarly, outpatient/clinic services, home-based care services and after-hours care need to be developed and strengthened to meet increasing patient loads and to strive for continuity of care regardless of patient location. For this, services need to function in unison and collaboratively with other medical specialities, aiming for integrated and parallel care [43].

Only 31 of 198 countries globally have achieved high-level (level 4 b) integration of palliative care into mainstream health service provision, with India rated at level 3a (isolated palliative care provision) in the most recent global mapping assessment. Integration and early referrals to palliative care will only be possible if the strategies are based on macro, meso and micro levels [44]. These include nationwide measures such as resource allocation, training and education of healthcare professionals, advocacy for patients and their families, the introduction of palliative care in healthcare educational curricula and more palliative care services in Indian hospitals. Increased involvement of the primary care team and other medical specialists in family meetings and palliative care consults and vice versa, and participation of the palliative care physicians in multidisciplinary meetings, may overcome some of the barriers to integration [45]. Palliative care needs to be seen as an essential service for the seriously ill rather than a competing speciality. The IAPC can initiate research to generate evidence on the integrated approach focusing on clinically relevant outcomes. Early integration of palliative care at the time of diagnosis of metastatic lung cancer is associated with a reduction in symptoms, improvement in quality of life and a smooth transition to hospice-based care [46]. The results have been consistent in other cancers as well [47], but are yet to gain sufficient evidence in non-cancer conditions like kidney failure, heart failure and chronic obstructive pulmonary disease [48]. This survey provides insight into areas for prioritisation.

**Figure 1.**
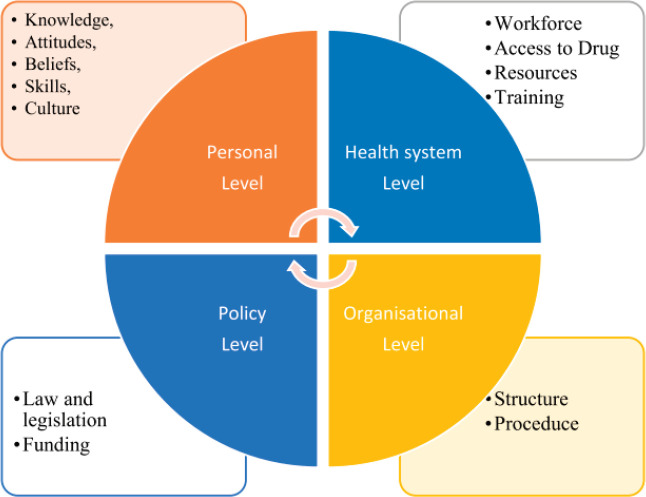
A modified socioecological framework [53].

Patients from lower-income groups across the country access government health services more than private hospitals. Merely 26% of participating centres were in the government sector. The non-government and private sectors are responsible for most of the palliative care services across the country. This is impressive from a financial standpoint, as healthcare expenses in India display the 70:70 paradox; 70% of expenses are out-of-pocket, of which 70% is spent on medications, leading to poverty and debt [49]. For the integration of palliative care into mainstream medical care, it is crucial to have an engagement in the state and central government health schemes, to bring about necessary changes in healthcare policy and increase the allocation of resources for palliative care [50]. The National Health Policy (2017) envisages a comprehensive primary health care package with geriatric, palliative and rehabilitative care. This is consistent with a public health model which is needed in LMICs [51]. Integrating care through national health care programmes like Ayushman Bharat, National Health Mission and Health and Wellness Centers would decrease out-of-pocket expenditure for socially disadvantaged groups. The socio-ecological model used by Abu Odah *et al* [52] to examine barriers to palliative care in LMICs reveals four domains that impact the provision (Figure 1).

Aligned with this socio-ecological model and in keeping with our findings, we support the suggested multipronged public health approach to drive improvement nationally: health policy (supporting the integration of palliative care and investment in systems of health care delivery); laws and policies (access to opioids); education and training of health professionals; methodologically rigorous and region-specific research and changes in the attitudes of society and health care professional towards palliative and end-of-life care [54]. The Standards tool takes its place as an instrument to map development and progress and to help services identify their priorities for improvement in a planned and focused way [55].

### Limitations

We identified several weaknesses in this project.

#### Representability of the centres:

We could not reach out to every palliative care centre in India but tried our best to contact the list in the centralised IAPC database. However, several new palliative care programmes have come up over the past decade, which are in the process of getting into the IAPC database. Capturing data from every such centre would need a bigger resource-intensive survey.

#### Lack of standardisation:

While a ‘gold standard’ set of indicators for a palliative care programme in India has not yet been achieved, the indicators used in this audit have been developed by leading experts in palliative care in India and internationally and endorsed by the IAPC. It would be beneficial to have a standardised definition of an operational palliative care programme describing the necessary infrastructure and staff qualifications. Repeating this national audit regularly will help track the progress of Indian palliative care development.

#### Survey questionnaire and methodology:

The survey questionnaire was developed from previously peer-reviewed research work [16]. Though we performed a face validity before adapting the survey items, future research could focus on rigorous psychometric methodology [56]. There were missing values, and a few items of the questionnaire were not answered by the participants initially, but telephone follow-up ensured that we obtained a complete dataset. ‘Sometimes’ seems to be the most chosen category in the survey responses. It has been seen that respondents in surveys tend to choose a middle-of-the-scale option, also known as central tendency bias when they are unsure of or lack the data to answer more specifically [57]. The scope of services was overlapping and not delineated. For example, 33% of centres had day-care services with only one of three modalities for treatment, i.e., surgery, chemotherapy or radiotherapy, but no inpatient beds dedicated to palliative care delivery, therefore, recorded no in-patient services. The results of this project may have recall bias [58], as information was collected through online forms and telephone calls.

### The way forward

The hospice and palliative care scenario in India has evolved significantly since the first hospice opened in the 1980s [59]. Progress has been slow and challenging, with multiple barriers faced at the personal, health system, policy and organisational levels. Challenges have brought about diversification and adaptation in the form of indigenous approaches like community-based palliative care (Neighbourhood Network in Palliative Care, NNPC) in Kerala, which has been adopted as a model for resource-poor countries around the world [36]. Indian palliative care services are heterogeneous and must evolve to suit the specific needs of their patients, drawing on evidence-based health systems data. Commitment, measurable targets and working partnerships between the Government of India, IAPC and other organisations supporting palliative care are needed to change the situation. We need better financing and integration of palliative care services into mainstream health care, expansion of service coverage, better training, evidence-based research, widespread recognition as a speciality practice, adoption of health technology and continuous quality improvement. Future research might also assess the quality of services relative to the ratio of the available work force and patient footfall in each of these centres. We also recognise that oncologists and other practitioners in peripheral hospitals provide supportive care to cancer patients and surveying these centres would provide an understanding of the quality of generalist palliative care in India.

## Conclusion

A detailed survey of 223 IAPC-registered centres identified specific and important gaps in service provision. To deliver essential standards, the centres must ensure regular and uninterrupted morphine, develop robust infrastructure, expand institutional service coverage, and home care programmes, and support their staff by providing training, self-care strategies and continuous professional development. Within institutions, better team health and integration with existing healthcare services and the wider community are needed. Training programmes are necessary to develop a multidisciplinary workforce.

## Key messages

Why this matters to me?

- Focussed efforts by individuals will help increase the quality and coverage of palliative care from category 2 (capacity building) to 4b (advanced stage of integration) [61]
- Modern health services and teams operate in silos, with organisations having great choice over the services they develop. This helps to develop services relevant to local needs

We need to work harder to address

- Workforce shortages – trained doctors and nurses
- Shortage of team members trained to deliver psychological, social and spiritual support
- Better assessment and documentation
- Uninterrupted supply of morphine
- Rigorous documentation of prescribed opioids
- Promotion of team health
- Inadequate ongoing professional education/training
- Community collaboration
- Funding

What are we doing well?

- Working towards access to ‘essential package for palliative care’
- Audit
- Research
- Institutional policy documents on pain management
- Institutional policy documents on end-of-life care

What can IAPC do?

- Define levels of care – so that teams can aspire to upgrade from ‘Basic services’ to ‘Centre of excellence’
- Emphasise the delivery of ‘Essential’ aspects of Palliative Care delivery
- Initiate and promote region specific research: service integration, evaluating indigenous solutions/local practices to guide policy and practice
- Provide technical support to new services for their development
- Strengthen national policy
- Work on regulatory requirements, oversight
- Work with National Accreditation Board for Hospitals and Healthcare Providers (NABH) for accreditation standards to ensure access and quality
- Work with the government for policy and health insurance funding

## Funding

The IAPC funded the research.

## Conflicts of interest

None declared.

## Figures and Tables

**Table 1.** Responses to the standards audit tool for Indian palliative care programmes.

Headers	Number (percentage)
***A. Type of services***	
Government hospital	59 (26.4)
Private hospital	141 (63.2)
Non-governmental organisation	11 (4.9)
Community	12 (5.4)
***B. Focus of work***	
Provide palliative care as and when an opportunity arises, but not the focus of the work	70 (31.4)
The focus of work is palliative care in cancer	68 (30.5)
The focus of work is palliative care in different settings (both malignant and non-malignant)	85 (38.1)

**Table:** 

Size of the programme	Number (percentage)	Average daily census of cases attended	The average length of service in days
***C. Descriptors***			
In-patient	151 (67.7)	29	9
Outpatient	223 (100)	56	Not applicable
Home-based care	134 (60.1)	17	33
Hospice	13 (5.8)	15	25
Cross consultation service	118 (52.9)	8	Not applicable

**Table:** 

*Part D: Essential criteria*						
The hospice/palliative care programme:						
	**Never (number/percentage)**	**Rarely (number/percentage)**	**Sometimes (number/percentage)**		**Often (number/percentage)**	**Always (number/percentage)**
Has a system in place for whole patient assessment, documentation and management that includes at a minimum						
Assessment and documentation of pain, pain scale	10	1	199		1	12
	4.5	0.4	89.2		0.4	5.4
Assessment and documentation of other symptoms	6	3	200		3	11
	2.7	1.3	89.7		1.3	4.9
Regular review of pain and other symptoms and titration of medications	9	1	191		9	13
	4.0	0.4	85.6		4.0	5.8
Provides access to essential medications as demonstrated by:						
An uninterrupted supply of immediate-release oral morphine	11	10	189		1	12
	4.9	4.5	84.7		0.4	5.4
Access to essential medicines and equipment (Essential Package)	2	19	1		3	198
	0.9	8.5	0.4		1.3	88.8
A system for documentation of step 3 opioids uses including names of patients and identification numbers, quantity dispensed each time and balance of stock after each transaction	1	1	199		9	13
	0.4	0.4	89.2		4.0	5.8
A Palliative service should adopt a team approach. It should have at least:						
Trained Doctor with a minimum of 10 days of clinical palliative care training under the supervision	2	10	190		9	12
	0.9	4.5	85.2		4.0	5.4
Trained Nurse with a minimum of 10 days of clinical palliative care training under the supervision	1	16	184		10	12
	0.4	7.2	82.5		4.5	5.4
Designated team members trained to deliver psychological, social and spiritual support	17	1	191		9	5
	7.6	0.4	85.6		4.0	2.2
The palliative care service engages the community and does not work in isolation						
There is evidence of interaction between the community and health care professionals in the establishment and ongoing operation of the services	1	3	197		14	8
	0.4	1.3	88.3		6.3	3.6
The palliative care service supports the health of the team through activities						
Regular team meetings	2	15	184		14	8
	0.9	6.7	82.5		6.3	3.6
Self-care training	1	20	192		4	6
	0.4	8.9	86.1		1.8	2.7
Debriefing	17	193	4		2	7
	7.6	86.5	1.8		0.9	3.1
The palliative care service has a programme of education and training						
Ongoing continuing professional education for the palliative care team	13	199	2		4	5
	5.8	89.2	0.9		1.8	2.2
Educational programmes on palliative care for fellow professionals	3	1	16		199	4
	1.3	0.4	7.2		89.2	1.8
***Part E: Desirable criteria***						
The hospice/palliative care						
Has sufficient access to free morphine (an essential package for poor patients)	1	17	193		1	11
	0.4	7.6	86.5		0.4	4.9
Provides home care services directly/indirectly	1	16	195		3	8
	0.4	7.2	87.4		1.3	3.6
Provides after-hours support directly/indirectly	9	1	200		4	9
	4.0	0.4	89.7		1.8	4.0
Has an institutional policy for pain management	6	1	11		194	11
	2.7	0.4	4.9		86.9	4.9
Has an institutional policy for end-of-life care	1	6	8		199	9
	0.4	2.7	3.6		89.2	4.0
Has access to ancillary services – dietetics, physical therapy, occupational therapy, physical rehabilitation	2	4	10		200	7
	0.9	1.8	4.5		89.7	3.1
Provides caregiver support including bereavement support	1	11	11		194	6
	0.4	4.9	4.9		86.9	2.7
Has significant contributions from volunteers	57	2	19		143	2
	25.6	0.9	8.5		64.1	0.9
Has the support of other health care professionals for palliative care work	15	1	4		199	4
	6.7	0.4	1.8		89.2	1.8
Conducts programmes to promote awareness, and advocacy for palliative care work through media support, Indian Medical Association, etc.	19	2	2		196	4
	8.5	0.9	0.9		87.9	1.8
The palliative care service fosters a healthy organisational culture which						
	Yes			No		
Regular team activities that foster team building	201			22		
	90.1			9.9		
Conflict resolution	213			10		
	95.5			4.5		
Administrators are supportive of palliative care	212			11		
	95.1			4.9		
The palliative care service has in place a programme of education and training						
Education programmes on palliative care for medical/nursing students/social work students	204			19		
	91.5			8.5		
Education programmes on palliative care for volunteers	198			25		
	88.8			11.2		
Awareness programmes on palliative care for the public	207			16		
	92.8			7.2		
Access to teaching material, textbooks and journals	210			13		
	94.2			5.8		
Participation in conferences and continuing medical education	204			19		
	91.5			8.5		
The palliative care service commits to continuous quality improvement						
Ongoing audit	212			11		
	95.1			4.9		
Participation in research	209			14		
	93.7			6.3		
The palliative care service participates in institutional activities						
Integration with mainstream care – participation in Journal Club, Ethics Committee, Multidisciplinary team meeting, tumour board, etc.	207			16		
	92.8			7.2		
Do you know of any other palliative care provider near you but not included in the list attached?	192			31		
	86.1			13.9		

## Footnotes

1 The Indian population is equivalent to 17.7% of the total world population

2 ‘Community-based’ services are based in the community, in the form of a partnership between the healthcare provider and members of the community who become partners in care delivery, which differs from institutional-based palliative care services with ‘community outreach’. The latter is more common in India (Organization 2021; Community engagement: a health promotion guide for universal health coverage in the hands of the people. Geneva: World Health Organization; 2020. Licence: CC BY-NC-SA 3.0 IGO) [60]
